# Biological Role of Unsaturated Fatty Acid Desaturases in Health and Disease

**DOI:** 10.3390/nu12020356

**Published:** 2020-01-29

**Authors:** Aleksandra Czumaj, Tomasz Śledziński

**Affiliations:** Department of Pharmaceutical Biochemistry, Faculty of Pharmacy, Medical University of Gdansk, Dębinki, 80-211 Gdansk, Poland; tomasz.sledzinski@gumed.edu.pl

**Keywords:** fatty acid desaturase, polyunsaturated fatty acid, desaturation, disease, FADS

## Abstract

Polyunsaturated fatty acids (PUFAs) are considered one of the most important components of cells that influence normal development and function of many organisms, both eukaryotes and prokaryotes. Unsaturated fatty acid desaturases play a crucial role in the synthesis of PUFAs, inserting additional unsaturated bonds into the acyl chain. The level of expression and activity of different types of desaturases determines profiles of PUFAs. It is well recognized that qualitative and quantitative changes in the PUFA profile, resulting from alterations in the expression and activity of fatty acid desaturases, are associated with many pathological conditions. Understanding of underlying mechanisms of fatty acid desaturase activity and their functional modification will facilitate the development of novel therapeutic strategies in diseases associated with qualitative and quantitative disorders of PUFA.

## 1. Introduction

Fatty acids (FAs) are essential for the normal functioning of all organisms. FAs are components of plasma membranes, function as energy storage material, and act as signal molecules regulating growth and differentiation of cells, as well as the expression of genes [1,2]. The structure of FAs can be modified by elongation and desaturation. The biological effects of FAs depend on the number of unsaturated bonds in their molecules [3,4].

The enzymes that insert unsaturated bonds to FA molecules are referred to as fatty acid desaturases. These enzymes catalyze the biosynthesis of monounsaturated fatty acids (MUFAs) and polyunsaturated fatty acids (PUFAs) by conversion of single bonds (C-C) into double bonds (C = C) in the acyl chain. Conversion of saturated FAs into MUFAs is catalyzed by stearoyl-CoA desaturase-1 (SCD1), but the role of this enzyme will not be discussed in this review. PUFAs are formed in the reactions catalyzed by another group of desaturases, unsaturated fatty acid desaturases. The type, expression level, and activity of unsaturated fatty acid desaturases determine the qualitative and quantitative composition of PUFA profile. These enzymes are present in all living organisms, but their role in bacteria, fungi, plants, and animals differ considerably. 

PUFAs, formed in the reactions catalyzed by unsaturated fatty acid desaturases, are important bioactive compounds that regulate many physiological processes. The most important PUFAs in this context seem to be omega-3 and omega-6 FAs, the names of which derive from the position of the first double bond from the methyl end. The group of omega-3 FAs includes alpha-linolenic acid (ALA, 18:3), eicosapentaenoic acid (EPA, 20:5), and docosahexaenoic acid (DHA, 22:6), whereas linoleic acid (LA, 18:2), gamma-linolenic acid (GLA, 18:3), dihomo-gamma-linolenic acid (DGLA, 20:3), and arachidonic acid (ARA, 20:4) are the examples of omega-6 FAs. PUFAs can produce various biological effects due to their ability to change the composition of plasma membranes, to regulate gene transcription, and to modulate cell signaling [5,6]. PUFAs are precursors of various lipid mediators, including pro- and anti-inflammatory eicosanoids and docosanoids. Prostaglandins, prostacyclins, and leukotrienes derived from PUFAs are involved in inflammatory reactions and immune response. ARA is a precursor of pro-inflammatory eicosanoids, whereas EPA and DGLA are substrates for the synthesis of anti-inflammatory eicosanoids. DHA is also a precursor of anti-inflammatory and immunomodulatory docosanoids, such as resolvins and protectins [7,8]. Considering their biological functions mentioned above, both omega-3 and omega-6 FAs are considered key factors in the prevention of some undesirable bodily reactions, such as autoimmune response. Moreover, PUFAs play a significant role in chronic diseases, including cardiovascular disorders, cancers, and diabetes mellitus [9,10].

In addition to controlling the PUFA content, recent studies have shown that unsaturated fatty acid desaturases play an important role in glycolytic nicotinamide adenine dinucleotide (NAD^+^) recycling in the cell and as a consequence, they can be an alternative route for the flow of reducing equivalents generated during glycolysis [11]. Under anaerobic conditions, when mitochondrial respiration is impaired, not only anaerobic glycolysis but also unsaturated fatty acid desaturases can oxidize the reduced form of nicotinamide adenine dinucleotide (NADH) to regenerate the NAD^+^ pool, which can be used by glyceraldehyde 3-phosphate dehydrogenase during glycolysis. Considering the complex function of PUFAs and unsaturated fatty acid desaturases itself (regardless from their products), changes in the expression or activity of fatty acid desaturases may have important consequences for the regulation of many processes taking place in the body, either those associated with basic metabolism or those involved in the development of various pathological conditions. In this review, we focus primarily on unsaturated fatty acid desaturases; we discuss the role of these enzymes, their effect on human health, potential therapeutic applications that have been proposed recently and future research possibilities.

## 2. Activity of Unsaturated Fatty Acid Desaturases

**Figure 1:**
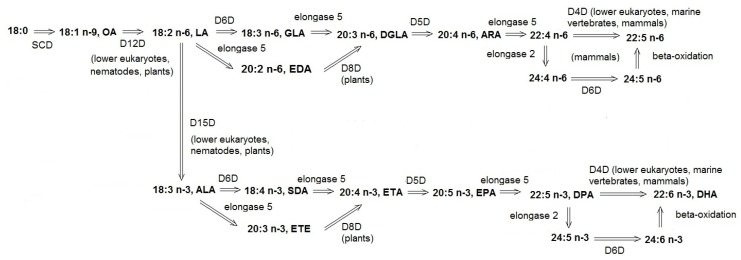
Desaturase-mediated synthesis of polyunsaturated fatty acids (PUFAs). ALA—alpha-linolenic acid, 18:3 n-3, ARA—arachidonic acid, 20:4 n-6, DGLA—dihomo-gamma-linolenic acid, 20:3 n-6, DHA—docosahexaenoic acid, 22:6 n-3, DPA—docosapentaenoic acid, 22:5 n-3, D4D—delta-4 desaturase, D5D—delta-5 desaturase, D6D—delta-6 desaturase, D8D—delta-8 desaturase, D12D—delta-12 desaturase, D15D—delta-15 desaturase, EDA—eicosadienoic acid, 20:2 n-6, ETA—eicosatetraenoic acid, 20:4 n-3, ETE—eicosatrienoic acid, 20:3 n-3, EPA—eicosapentaenoic acid, 20:5 n-3, GLA—gamma-linolenic acid, 18:3 n-6, LA—linoleic acid, 18:2 n-6, OA—oleic acid, 18:1 n-9, SCD—stearoyl-CoA desaturase (delta-9 desaturase), SDA—stearidonic acid, 18:4 n-3.

Desaturase genes are widely spread in plant and animal kingdoms [12,13,14]. Fatty acid desaturases convert MUFAs to PUFAs or insert additional unsaturated bonds into already existing PUFAs, but in some organisms not all of these activities are present (Figure 1). A plethora of various fatty acid desaturases have been identified, each of them inserting the double bond into a unique position. The regional selectivity of desaturases allows them to distinguish between the two ends of the FA acyl chain. A given desaturase always inserts the new double bond between the already existing unsaturated bond and the methyl end (methyl-end desaturase, e.g., delta-12 or delta-15 desaturase) or carboxyl end (front-end desaturase, e.g., delta-4, delta-5, delta-6 desaturase). The name of the given enzyme reflects the position of the acyl chain in which the double bond is inserted. According to a traditional naming convention, an enzyme from this group is referred to as ‘delta X desaturase’, where X is the number corresponding to the position from the molecule’s end at which the double bond is inserted, whereas delta (Δ) means that the positions are counted from the carboxyl end.

The reaction catalyzed by desaturase is an aerobic process. Aside from the presence of oxygen (as a hydrogen acceptor), reaction requires cytochrome b5 (electron carrier) and NADH-dependent cytochrome b5 reductase. During the first stage of the desaturation process, reduced cytochrome b5 interacts with the non-heme iron active site of the desaturase, which enables the enzyme to react with O_2_ and a substrate. This results in the activation of hydrogen at the carbon of incipient double bond in substrate. The product of this relatively slow stage is a very short-lived carbon-centered radical [15,16]. During the second stage, this transient molecule loses the second hydrogen via rapid disproportionation, which leads to the formation of the final product with a new double bond, along with two molecules of water [17].

Eukaryotic desaturases share similar structural motifs. These enzymes have an N-terminal cytochrome b5 domain, which contains the heme-binding motif, crucial for catalytic activity of desaturases [18,19]. The C-terminal domain contains three histidine motifs with seven histidines (the so-called His box) which are also vital for the catalytic activity. Substitution of histidine with glutamine in the third motif from the C-end is a specific feature of all desaturases that insert a double bond between the already existing bond and carboxyl end. Separation of the first and second histidine motif by a long histidine fragment, aside from fatty acid desaturases, is also observed in aldehyde hydroxylases and carboxylases [20,21].

The most well-known examples of fatty acid desaturases are delta-6 desaturase (D6D), delta-5 desaturase (D5D), delta-12 desaturase (D12D), and delta-15 desaturase (D15D). D6D catalyzes the insertion of the double bond between sixth and seventh carbon from the carboxyl end. Reaction of D6D is the rate-limiting step in the formation of GLA from LA and stearidonic acid (SDA, 18:4 n-3) from ALA [22]. D6D can use at least two other substrates, tetracosatetraenoic acid (24:4 n-6) and tetracosapentaenoic acid (24:5 n-3), converting them into tetracosapentaenoic acid (24:5 n-6) and tetracosahexaenoic acid (24:6 n-3), respectively. Aside from the activity of delta-6 desaturase, this enzyme can also show other desaturase activities, e.g., synthesize DGLA and eicosatetraenoic acid (ETA, 20:4 n-3) from eicosadienoic acid (EDA, 20:2 n-6) and eicosatetraenoic acid (ETE, 20:3 n-3), respectively [23,24]. Moreover, D6D is responsible for the synthesis of sapienic acid, the most abundant FA of human sebum, catalyzing desaturation of 16:0 to 16:1 n-10. The latter reaction is a unique example of this enzyme activity because it leads to the synthesis of MUFA, and this reaction occurs exclusively in sebaceous glands [25]. D5D inserts double bond at the fifth position from the carboxyl end. This enzyme is primarily involved in the synthesis of ARA and EPA, using DGLA and ETA, respectively, as the substrates [26]. Other acids, such as 20:3 n-3 and 20:2 n-6, can also serve as the substrates for D5D. Moreover, some isoforms of D5D can catalyze the synthesis of atypical products, such as non-methylene-interrupted fatty acids (FA where double bound are interrupted with group other than methylene), e.g., 18:3 Δ5, 9, 12 and 20:4 Δ5, 11, 14, 17 [19,21,27,28]. D12D and D15D are the key enzymes involved in the synthesis of omega-3 and omega-6 FAs. D12D is responsible for the conversion of oleic acid (OA, 18:1 n-9) to LA, and D15D desaturates LA to ALA [29]. These enzymes are not expressed in all mammals, including humans; as a result, they cannot synthesize LA and ALA de novo and need to obtain these essential FAs from the diet. Delta-12 and/or delta-15 desaturases are expressed in lower eukaryotes and most plants, as well as in some animals, such as cockroaches, house crickets, and parasitic wasps [30,31,32].

Regulation of the expression and activity of unsaturated fatty acid desaturases is a very complex biochemical phenomenon. Dietary PUFA intake and transcriptional regulation are well-recognized determinates of unsaturated fatty acid desaturases activity. Many studies proved that dietary FA can influence gene expression of unsaturated fatty acid desaturases [33,34]. Both D5D and D6D expression were reduced by PUFAs in multiple experimental models, ranging from cell lines cultured in vitro and treated with different PUFAs to animals fed with PUFA-enriched diets (including grouper larvae, European sea bass, mice, piglets, and baboons) [35,36,37,38]. Unsaturated fatty acid desaturases gene expression is also regulated by insulin and gonadal hormones [39,40,41]. Several transcription factor binding site motifs were identified in promoters regions of unsaturated fatty acid desaturases, including nuclear factor Y (NF-Y), CCAAT enhancer binding protein (C/EBP), sterol regulatory element (SRE), nuclear factor 1 (NF-1), stimulatory protein 1 (Sp1), activated protein 1 (AP1), hepatocyte nuclear factor 4α (HNF4α), and peroxisome proliferator activated receptor γ (PPARγ) [42,43,44]. Epigenetic alterations (i.e., methylation of DNA) may also contribute to changes in desaturase activity [45,46]. Furthermore, recent findings suggest that D5D and D6D activity can change in response to the cytosolic NAD^+^/NADH ratio. The decreased cytosolic NAD^+^/NADH ratio (for example, as a result of inhibition of aerobic cellular respiration) increases the activity of unsaturated fatty acid desaturases, without change in mRNA or protein levels [11].

## 3. Role of Unsaturated Fatty Acid Desaturases

The activity of desaturases determines, together with dietary FA intake, the qualitative and quantitative composition of unsaturated FAs. Products of these enzymes are essential components of all biological systems. The exact role of desaturases depends on the organism in which they are expressed.

### 3.1. Role of Unsaturated Fatty Acid Desaturases in Microorganisms

Firstly, the desaturases genes were discovered, and their functions were recognized in microorganisms. For example, the first D6D gene was cloned from cyanobacterium *Synechocystis*, the first D5D gene was cloned from fungus *Mortierella alpina*, and the first D8D gene was cloned from the protist *Euglena gracilis*. Up to now, microorganisms remain a subject of interest in many researches on the structure, function, and activity of unsaturated fatty acid desaturases [47,48,49].

In bacteria, changes in the PUFA composition in plasma membranes can be helpful in the adjustment to unfavorable environmental conditions. These organisms can change the activity of desaturases to adapt the PUFA content to changing temperature, pH, and atmospheric pressure to maintain appropriate fluidity of plasma membranes. Long-chain PUFAs are detected in the largest amount in bacteria inhabiting cold marine environments, such as polar regions or deep seas. Transfer of such bacteria into a warmer environment has resulted in changes in expression of desaturases genes and in a decrease in the PUFA content in membrane phospholipids [50,51,52,53].

PUFAs in microorganisms usually have 16 or 18 carbons, nevertheless, some microorganisms were shown to synthesize ARA, EPA, and DHA [54]. Nowadays primary sources of EPA and DHA in our diet are marine water fish species. However, their availability is limited due to catch limits implemented to prevent resource depletion. Hence, the use of microorganisms expressing specific homologous or heterologous unsaturated fatty acid desaturases capable of synthesizing EPA and DHA seems to be a promising alternative source of omega-3 FAs [54,55,56,57]. Current commercially used microorganisms to produce n-3 PUFA, such as *Crypthecodinium cohnii*, *Schizochytrium* sp., or *Nannochloropsis* sp., are the result of intensive screening and selective breeding procedures. Genetic engineering of unsaturated fatty acid desaturases in microorganisms opens up new possibilities to maximize the efficiency of upstream and downstream processing [58,59,60,61].

### 3.2. Role of Unsaturated Fatty Acid Desaturases in Plants

FA composition is crucial for the growth and vegetation of plants [62]. FA desaturation is a key factor determining the tolerance of plants to various environmental stressors [63]. Enhanced accumulation of PUFAs, due to changes in desaturases gene expression level, is postulated to facilitate cold adaptation, maintaining normal fluidity and integrity of plasma membranes [64]. For example, in many plants the expression of FAD8 gene (genes encoding fatty acid desaturases in plants belong to the FAD family) is strongly induced by low temperatures (product of this gene catalyzed by the conversion of diene fatty acids to triene fatty acids), [64,65]. However, excessive accumulation of PUFAs may also exacerbate a thermal injury [66]. Transgenic tobacco transformed with *FAD7* gene showed greater resistance to cold, whereas the plants with silenced *FAD7* contained less trienoic fatty acids and showed better tolerance to high temperatures than the wildtype plants [67,68]. Furthermore, *FAD2* and *FAD6* genes in seedlings were shown to be activated by salt and osmotic stress [69]. Mutants with *FAD2* deficiency accumulated more sodium ions (Na+) ^+^ in the cytoplasm of root cells and were highly sensitive to salt stress during seed germination and early seedling growth [70].

The activity of various fatty acid desaturases is also crucial in the context of nutritional properties of edible oils, as these enzymes determine the contents of individual PUFAs in the final product [71,72]. Progress in genetic engineering opened a perspective for modification of fatty acid desaturases to enrich cultivated plants in particularly desirable FAs. Many research groups obtained transgenic plants that can synthesize and accumulate selected FAs, such as OA, EPA, DHA, and SDA [19,73,74,75,76,77]. For example, expression of borage (*Borago officinalis*) D6D in oilseed crops contributed to increase in SDA levels up to 20% of all FAs present in these plants [73,74]. High levels of EPA were reported in Ethiopian mustard (*Brassica carinata*) after its transformation with the fungal D5D gene [78]. Few research groups were able to produce DHA in plants by using the D4D gene from microorganisms [79,80,81,82]. A higher specific PUFA consumption is recommended by doctors and nutritionists in several diseases, such as cardiovascular diseases, diabetes, and inflammatory disorders. However, the efficacy, quality, and safety of PUFA supplements available on the market are questionable because they are beyond any pharmaceutical control [83,84,85]. Several studies conducted in geographically diverse locations have shown that vegans and vegetarians have lower levels of DHA and EPA in the serum compared to omnivores [86,87,88]. Therefore, the use of plants with modified fatty acid desaturases seems to be a promising alternative for dietary n-3 PUFA consumption. This can be especially important for vegan and vegetarian mothers during breastfeeding.

### 3.3. Role of Unsaturated Fatty Acid Desaturases in Humans and Other Mammals

Our unsaturated fatty acid desaturases activity and our PUFA profile is a consequence of diet and genetic makeup. Even one single nucleotide polymorphisms (SNPs) in *FADS* genes (mammalian desaturase genes that belong to the FADS family) were shown to be associated with altered activity of fatty acid desaturases and significant changes in plasma lipid profile [89,90,91,92,93]. People with high dietary intake of ALA who carried one or two altered *FADS1* alleles (the so-called rs174546 variant), presented with lower concentrations of total cholesterol and non-HDL cholesterol than those with other allelic variants and the same intake of this FA (*FADS1* encodes D5D) [94]. An exploratory study from Australia reports that *FADS*1 genotype can also alter the effects of long-chain PUFA supplementation. Only children with certain *FADS1* SNPs benefit from fish oil supplementation [95]. Recent studies of the mouse *FADS2* gene (encodes D6D) demonstrated that even one non-synonymous SNP can alter properties of the enzyme. SNP A→G was associated with the change of amino acid in the region responsible for iron binding. The presence of G allele seems to be associated with lower activity of the desaturase since mice carrying this allele had higher levels of LA and ALA and lower levels of ARA, EPA, and DHA. Moreover, the G allele was shown to be more common in mice with higher basal metabolic rate [96]. The protein encoded by the third mammalian fatty acid desaturase gene, *FADS3*, has yet not been ascribed any specific function. Expression of *FADS3* was found in many human organs. Surprisingly, recent in vitro studies demonstrated that the product of *FADS3* in rat encodes the enzyme which can catalyze delta-13 desaturation of trans-vaccenic acid (VA, trans-11 18:1) to trans-11, cis-13 conjugated linoleic acid (CLA; trans-11, cis-13 18:2). Hence, *FADS3* might be the first gene encoding methyl-end desaturase in mammals, but this concept still has not been confirmed in vivo [97,98]. What is interesting, some studies have shown that unsaturated fatty acid desaturases activity resulting from genetic variants of *FADS* genes can be masked. Alterations with the gene-related PUFA profile was only observed in normal weight women, whereas overweight/obese women (with a BMI ≥ 25) are less affected by FADS genetic variants in this regard [99]. Exploration of gene-BMI interactions in the general population or male subpopulation is still needed.

Fatty acid desaturases seem to exert a variable effect on human health. This functional link results from a relationship between specific polymorphisms of desaturase genes and various metabolic phenotypes (Table 1) [100]. Many studies demonstrated a relationship between the activity of these enzymes and various complex diseases, such as coronary artery disease (CAD). Among patients with CAD, the activity of D6D was higher in comparison to healthy subjects. Moreover, specific SNP can alter the probability of CAD occurrence. For example, SNP in position rs174537G/T is associated with a higher risk of CAD. Variant rs174537T is associated with a lower risk of CAD, while variant rs174537G is more common in CAD patients [101,102,103]. Fortunately, there is an evidence that appropriate modification of PUFA intake from the diet can prevent the unfavorable effects of *FADS* polymorphism [104].

Since n-3 PUFA are considered as anti-inflammatory substances, altered PUFA metabolism facilitates the chronic inflammation observed in Crohn’s disease (CD). Data from the genome-wide association study point to a few SNP in *FADS* genes associated with CD risk [167,168]. Analysis of D6D in mesenteric adipose tissue biopsies (abnormal mesenteric adipose tissue is the hallmark of CD) shows a lower level of D6D in CD specimens than in normal tissue at both mRNA and protein level [169]. However, the activity of D6D, calculated as enzyme activity indexes based on the fatty acid concentration in plasma, was significantly higher in the CD patients than in the healthy volunteers [170]. Moreover, studies suggest that patients with CD benefit from varying degrees of n-3 PUFA supplementations [171]. Lack of consistency across studies may be explained by the presence of gene-diet interactions. For example, patients with specific *FADS* variants associated with higher endogenous production of n-6 PUFA have a greater risk of developing CD even with lower dietary intake of n-6 PUFA, so they can benefit from n-3 PUFA supplementation, whereas patients with *FADS* variants associated with a lower endogenous production of n-3 PUFA, even with high dietary PUFA intake, still tends to have a lower n-3 PUFA level, so they benefit less from n-3 PUFA supplementations than patients with other *FADS* variants [168,172,173]. This suggests that in the future, a more personalized approach is needed. 

Many studies conducted in humans and mice showed correlations between the activities of delta-5 and delta-6 desaturases and metabolic syndrome, insulin resistance, and obesity. They reported that there is a strong positive correlation between D6D activity and the risk of these conditions, whereas the activity of D5D is inversely related to metabolic syndrome, insulin resistance, and obesity (no genetic backgrounds were investigated in these studies) [174,175,176]. Comparative genetic analysis of persons with insulin resistance and normal sensitivity to insulin demonstrated downregulation of D5D expression in adipose tissue and muscles of the former group (no activity measurements were made in this study) [177]. The problem is even more complicated if we take into consideration the fact that certain SNP can modulate desaturases activity. Some studies from Japanese populations show that some variant of *FADS1* (SNP rs174550) was significantly associated with an increased risk of type 2 diabetes [178]. Interestingly, the same SNP in the European population was associated with the lower fasting plasma glucose level in a normal physiological range, but not with pathological glucose levels [179]. Data from multiple experiments on mice suggest that the development of selective desaturases inhibitors can be beneficial in the treatment of human diabetes, obesity, and atherosclerotic cardiovascular disease [180,181,182]. Mice with D6D knockdown developed resistance against high-fat diet induced obesity, so maybe some metabolic benefits for obese people can be gained with D6D inhibitors [183]. Since obesity is considered as a chronic low-grade inflammation state, D5D inhibitors can be also beneficial. Potential D5D inhibitor can increase DGLA level (precursor of anti-inflammatory eicosanoids) and decrease AA level (precursor to pro-inflammatory eicosanoids). Insulin resistance is a complicated condition and several factors including genetic makeup, lifestyle, and diet contribute to its development. Considering them separately may lead to inconsistencies across different studies, such as in case of studies on the effectiveness of PUFA supplementation. Studies have shown, that n-3 PUFA supplementation can be effective in prevention or reversion of insulin resistance, but only together with reducing the intake of saturated FA, trans-FA, and n-6 PUFA. Supplementation of n-6 PUFA may also decrease insulin resistance but only if n-6 PUFA replaces SFA, trans-FA, or sucrose. Replacing n-3 PUFA with n-6 PUFA in the diet can worsen insulin resistance [184,185,186]. n-3 PUFA may prevent insulin resistance by a number of different mechanisms, including regulation of inflammation, modulation of adiponectin and leptin secretion, and influencing the expression of several genes involved in carbohydrate and lipid metabolism [187,188,189]. It seems that associations between desaturases and complex metabolic conditions such as metabolic syndrome, insulin resistance, and obesity are still not fully understood and some mechanisms still remain elusive.

Patients with non-alcoholic fatty liver disease (NAFLD) present a higher level of free FA in the serum and altered profile of FA in comparison to healthy subjects, including higher total SFA, higher total MUFA, lower total PUFA [190,191]. Available evidence shows that altered activity of fatty acid desaturases is one of the risk factors of NAFLD. Patients with NAFLD were shown to present with a higher activity of D6D and lower activity of D5D than healthy volunteers [192,193,194]. A similar observation was made in the case of pediatric non-alcoholic liver disease [195]. Differences in activity of desaturases are often connected to naturally existing genetic variations of those genes [196]. Better understanding of the connection between locus polymorphism and level of functional enzyme can result in the personalized treatment strategy for NAFLD in the near future. A pilot study from 2018 has shown that NAFLD patients with alleles connected to a low D5D activity benefit more from n-3 PUFA supplementation (significant reduction in steatosis, fibrosis, ballooning, and NAFLD activity score) than patients with others SNP variants [195]. So far, SNPs in patatin-like phospholipase domain-containing protein 3 (PNPLA3) and transmembrane 6 superfamily member 2 (TM6SF2) regions have been identified and validated in large cohorts of patients as a biomarker for NAFLD risk. Maybe in the near future the SNPs in *FADS1* will be the third biomarker for NAFLD but some large-scale research is still needed [197,198]. However, the situation is complicated by the fact that in NAFLD, as well as, in CAD disturbances in unsaturated fatty acid desaturases are observed and NAFLD is often accompanied by CAD. What is more, CAD is one of the leading causes of death in this group of patients. Nevertheless, there is almost no research on the associations among NAFLD, CAD, and FADS polymorphism [199]. A deeper understanding of FADS SNP in the context of NAFLD with CAD is essential if we want to use a genetic background for early diagnosis and prevention.

Unsaturated fatty acid desaturases also play a significant role in carcinogenesis, including cancer cell survival, metastasis, and drug resistance [200,201]. Genome-wide association study in East Asians population identifies a loci map to *FADS1* and *FADS2* genes as associated with colorectal cancer and laryngeal squamous cell carcinoma risk [109,202]. Unfortunately, there is no information on this matter from populations from other continents, so it is not clear if this can be treated as a universal prediction marker. Moreover, in patients with malignant melanoma, breast, brain and lung malignancies, expression and activity of D6D in cancer tissues turned out to be higher than in adjacent normal tissues [203,204]. It cannot be excluded that this is a common feature of all malignant neoplasms [205]. In animal studies, inhibition of delta-6 desaturase (both through knockdown and via RNAi) effectively prevented cancer growth. Since D6D is an enzyme catalyzing the rate-limiting step of AA synthesis, its activity is crucial for the production of pro-inflammatory eicosanoids, downstream metabolites of AA, such as prostaglandins, leukotrienes, and epoxy-eicosatrienes. Hence, it seems logical that inhibition of the principal metabolite (AA) formation might be a more effective way to reduce the synthesis of pro-inflammatory eicosanoids than blocking each specific pathway (i.e., cyclooxygenase, lipoxygenase, and cytochrome P450 epoxygenase pathway), separately [206]. Reduced unsaturated fatty acid desaturases activity can have an anti-proliferative effect not only because of reduced eicosanoid availability for cell signaling. Another possibility is the altered cytosolic NAD^+^/NADH ratio, which can modulate glycolysis and lactate fermentation—two important sources of respiratory fuel in cancer cells [207]. Studies have shown that transient knockdown of D5D and D6D in cells cultured in vitro resulted in changes in the reactive oxygen species (ROS) level, pyruvate consumption, and cell proliferation [11]. However, further studies are required to resolve whether unsaturated fatty acid desaturases-mediated NAD^+^/NADH ratio play an important role in the Warburg effect in cancer cells. Taking all information together, fatty acid desaturation can be a new metabolic marker and therapeutic target in certain types of cancer. However, according to the authors’ knowledge, no *FADS1* or *FADS2* inhibitors in cancer treatment are undergoing clinical trials.

Fatty acid desaturases also play an important role during pregnancy and breastfeeding. Maternal levels of ARA, EPA, and DHA influence the amount of these acids delivered to the fetus directly or provided with maternal milk after birth. Hence, desaturase activity has an impact on the qualitative and quantitative composition of n-3 and n-6 FAs delivered to the child. FAs, among them ARA, EPA, and DHA, play a key role in growth, development of neurons, and functioning of the immune system in infants [108,180,208,209,210]. Moreover, the development of cognitive function and intellectual development of the child may be determined by a unique composition of FAs contained in maternal milk [211,212]. Interestingly, few studies also mention a relationship between breast milk fatty acids composition and postnatal HIV-1 transmission. ALA can be elongated to create ETE or desaturated to create SDA. When D6D is less active than elongase 5 the amount of ETE in breast milk increases. A higher concentration of ETE was associated with a lower risk of HIV-1 transmission [213]. Unfortunately, a large prospective cohort study is still lacking in this matter.

FAs, especially n-3, can modulate behavior and cognitive function. Animals maintained on a diet excluding or restricting substrates for fatty acid desaturases showed greater locomotor hyperactivity and worse cognitive skills. It was observed that children with specific SNPs in *FADS* genes (so called DHA-increasing cluster) score more in personal and social skills questionnaire [214]. Moreover, studies demonstrated a link between the variability of nucleotide sequences within desaturase encoding genes and the incidence of attention-deficit hyperactivity disorder (ADHD) [215].

The type of FAs contained in membrane phospholipids may modulate a broad range of biological mechanisms and pathways in the brain, including neurons, glial cells, and endothelial cells integrity and survival, neurotransmission (dopaminergic, serotonergic, glutamatergic, and cholinergic), and neuroinflammation, and [216,217,218]. PUFAs, especially ARA and DHA, are the major constituents of the neuronal membranes. However, the cause and effect relationship between mental disorders and unsaturated fatty acid desaturases is still unclear. Patients with neuropsychiatric disorders, such as bipolar affective disorders and schizophrenia often present overexpression and increased activity of D6D and at the same time, the levels of n-3 PUFA (DPA, DHA) and n-6 PUFA (EDA, ARA, 22:4 n-6) in neuronal membrane phospholipids were decreased [219,220,221]. Evidence from in vitro animal and clinical studies suggest that long-term treatment with risperidone (an antipsychotic drug) upregulate the expression and activity of multiple lipogenic genes, including D6D. Interestingly, some studies in animal models suggest that prenatal dietary PUFA deficiency (especially ARA and DHA) may affect early neurodevelopment of the offspring and thereby predispose to the development of schizophrenia [222]. Mice that had experienced gestational and early postnatal dietary ARA and DHA deprivation have shown schizophrenia-like phenotypes at adulthood (lowered levels of motivation, depressive symptoms, and impaired cognitive functions). Since the PUFA profile can be determined both by dietary PUFA intake and desaturase activity, it seems reasonable to assume that maternal activity of desaturases may also play a role in the risk of schizophrenia in offspring. A few studies on rats from the late 1970s and early 1980s mentioned changes in maternal, fetal and pups D5D and D6D activities in response to mothers’ low essential FA diet, nevertheless, until this day there are no studies on the relationship between mothers’ unsaturated fatty acid desaturases (activity and/or genetic variants) and schizophrenia risk in the offspring [223,224]. Furthermore, a relatively small number of studies have examined the therapeutic utility of PUFA supplementation in schizophrenia and none of them mentioned the potential use of *FADS* inhibitors. Moreover, results of these studies are inconsistent. Observed effects of PUFA supplementation vary from a significant increase in severity of schizophrenia symptoms, through no major effect on symptoms severity to decrease the intensity of symptoms and improve the level of patients functioning [225,226,227].

## 4. Conclusions

Desaturation of FAs plays a key role in the biosynthesis of lipids. In addition to dietary PUFA intake, the activity of fatty acid desaturases is one of the main determinants of the qualitative and quantitative profile of PUFAs. Changes in the PUFA profile may be either beneficial (e.g., facilitate survival under unfavorable environmental conditions) or harmful, resulting in the development of various pathologies. Further research on fatty acid desaturases will provide a better insight into the metabolic effect of the PUFA profile on human health. Understanding of these relationships might contribute to the development of novel therapeutic strategies based on modifications of fatty acid desaturase activities.

## Figures and Tables

**Table 1:** Selected traits associated with unsaturated fatty acid desaturases genetic variants—(‘?’- SNP variants not specified in the study).

Type of Desaturases	SNP Variants	Associated Trait	Nature of The Change	Tested Population	Reference
FADS1	rs174551-?	Alanine transaminase levels	Higher serum ALT level	East Asian	[105]
	rs174541-C	Bipolar disorder	Specific variant more common in subjects with the disease	East Asian, European	[106,107]
	rs174556-T	Breast milk fatty acid composition	Higher breast milk AA level	South Asian	[108]
	rs174549-A	Cancer (Laryngeal squamous cell carcinoma)	Specific variant more common in subjects with the disease	East Asian	[109]
	rs174564-G rs174546-T rs174549-A rs174547-C	Cardiology traits	Higher pulse Shorter QT interval Increased heart rate Lower resting heart rate	European, African, Hispanic/Latin American, Asian unspecified, Native American	[110,111,112,113]
	rs174546-C rs174550-T rs174551T rs174547-C rs174546-T	Cholesterol	Higher level of HDL Higher total cholesterol Higher level of LDL Lover level of HDL Lower total cholesterol Lower level of LDL	European, East Asian, South Asian, Hispanic/Latin American, African American/Afro- Caribbean, Oceanian, Native American	[114,115,116,117,118,119,120,121,122,123,124,125,126,127]
	rs174550-T	Fasting blood glucose	Increased fasting blood glucose	Hispanic/ Latin American, African American/Afro- Caribbean, East Asian, Oceanian, Native American, European	[123,128,129,130]
	rs174548-G rs174549-A	Fatty acid desaturase activity	Not specified Lower activity	East Asian, European	[91,131,132]
	rs174549-A rs174548-Grs174555-C rs174548-G	Hematology traits	Higher monocytes % Lower white blood cell count Higher platelet count, Sum eosinophil basophil counts Lower granulocytes count Lower red cell distribution width	European, East Asian	[133,134,135,136,137]
	rs174547-T	Height	Unspecified	East Asian, European	[133,138]
	rs174550-T rs174547-T	Plasman-3 polyunsaturated fatty acid level	Higher EPA level Lower ALA level Higher DPA level	European	[139]
	rs174550-T rs174547-C rs174546-T	Plasman-6 polyunsaturated fatty acid levels	Higher adrenic acid Lower ARA level Lower DGLA level	European, East Asian	[131,140]
	rs174546-T	Triglyceride levels	Higher TG level	European, Hispanic/ Latin American, African American or Afro-Caribbean, South Asian, East Asian, Oceanian, Native American	[105,115,116,117,118,119,120,121,123,124]
FADS2	rs174566-A rs174621-G	Asthma	Specific variant more common in subjects with the disease	European	[141]
	rs174592-G rs174581-A	Balding (type 1, male-pattern baldness)	Specific variant more common in subjects with the disease	European	[133,142,143]
	rs12226877-A rs28456-G rs174576-A	Bipolar disorder	Specific variant more common in subjects with the disease	East Asian, European	[106,107]
	rs174594-A rs1535-A rs2072113-C	Cancer (Laryngeal squamous cell carcinoma, Colorectal cancer, lung cancer)	Specific variant more common in subjects with the disease	East Asian, European, African American/Afro- Caribbean	[109,144,145,146,147]
	rs174577-A rs174564-G rs174583-T rs174577-?	Cardiology traits	Shorter P-wave duration Higher pulse pressure Shorter QT interval Shorter QRS duration	South Asian, European	[111,112,113,148,149,150]
	rs174570-G rs174577-C rs174566-G rs174570-T	Cholesterol levels	Higher total cholesterol, HDL, LDL levels Higher HDL level Higher LDL level Lower LDL level	European, Hispanic/Latin American, African American or Afro- Caribbean, East Asian	[114,115,116,118,119,120,121,122,125,151,152]
	rs174583-T rs174577-A	Comprehensive strength and appendicular lean mass	Higher comprehensive strength and appendicular lean mass	East Asian	[153]
	rs174566-G rs2072113-T	Fatty acid desaturase activity	Decrease activity	European, East Asian	[91,131,132]
	rs174601-T	Gondoic acid (20:1 n-9) levels	Higher FA level	East Asian, European	[154]
	rs968567-T rs2727271-? rs174570-C rs174577-A rs61897795-G rs2727271-T	Hematology traits	Higher IgA level Lower albumin-globulin ratio Higher glycated hemoglobin level Higher transferrin level Lower neutrophil count Higher non-albumin protein levels	East Asian, South Asian, European	[105,133,134,136,155,156,157]
	rs174574-A	Heel bone mineral density	Higher bone mineral density	European	[158,159]
	rs174599-?	Hypothyroidism	Lower thyroid hormones	European	[133]
	rs4246215-T	Inflammatory bowel disease	Specific variant more common in subjects with the disease	European	[160,161]
	rs174574-A rs1535-A	Plasma n-3 PUFA levels	Lower level of EPA, Lower ALA level Higher DPA	European	[139]
	rs174577-C rs2727270-T rs174578-T	Plasma n-6 PUFA levels	Higher ARA level Higher LA level Lower LA level	European, East Asian	[131,140]
	rs968567-C	Rheumatoid arthritis	Specific variant more common in subjects with the disease	European, East Asian, African American Afro-Caribbean	[162,163]
	rs174560-C	Sleep duration	Longer habitual sleep duration	European	[164]
	rs174564-G rs174577-C	Triglyceride levels	Lower TG level	European, Hispanic/ Latin American, African American/ Afro-Caribbean, South Asian, East Asian	[105,115,116,117,118,119,120,121,123,124]
FADS3	rs1000778-A	Sphingolipid levels	Lower sphingolipids level	European	[165]
	rs174468-A rs174448-A	Plasma n-3 PUFA levels	Higher level of ALA Lower level of DPA, EPA	European	[139]
	rs174449-A rs174448-A	Trans fatty acid levels	Higher concentrations of cis/trans-18:2	European, African American/Afro- Caribbean, East Asian, Hispanic/Latin American	[139,166]

## Abbreviations

ADHD	Attention-Deficit Hyperactivity Disorder
ALA	alpha-linolenic acid, 18:3 n-3
ARA	arachidonic acid, 20:4 n-6
CAD	coronary artery disease
CD	Crohn’s disease
CLA	conjugated linoleic acid, trans-11, cis-13 18:2
D5D	delta-5 desaturase
D6D	delta-6 desaturase
D12D	delta-12 desaturase
D15D	delta-15 desaturase
DGLA	dihomo-gamma-linolenic acid, 20:3 n-6
DHA	docosahexaenoic acid, 22:6 n-3
DPA	docosapentaenoic acid, 22:5 n-3
EDA	eicosadienoic acid, 20:2 n-6
ETA	eicosatetraenoic acid, 20:4 n-3
ETE	eicosatrienoic acid, 20:3 n-3
EPA	eicosapentaenoic acid, 20:5 n-3
FA	fatty acid
GLA	gamma-linolenic acid, 18:3 n-6
LA	linoleic acid, 18:2 n-6
MUFA	monounsaturated fatty acids
NAD	nicotinamide adenine dinucleotide
NADH	reduced nicotinamide adenine dinucleotide
NAFLD	non-alcoholic fatty liver disease
OA	oleic acid, 18:1 n-9
PUFA	polyunsaturated fatty acids
SCD	stearoyl-CoA desaturase
SDA	stearidonic acid, 18:4 n-3
SNP	single nucleotide polymorphism
VA	trans-vaccenic acid, trans-11 18:1
